# Killing two birds with one stone: how intervening when witnessing bullying at the workplace may help both target and the acting observer

**DOI:** 10.1007/s00420-020-01575-w

**Published:** 2020-09-14

**Authors:** Morten Birkeland Nielsen, Michael Rosander, Stefan Blomberg, Ståle Valvatne Einarsen

**Affiliations:** 1 National Institute of Occupational Health, Pb. 8149 Dep, 0033 Oslo, Norway; 2 Department of Psychosocial Science, University of Bergen, Bergen, Norway; 3 Department of Behavioural Sciences and Learning, Linköping University, Linköping, Sweden; 4 Department of Clinical and Experimental Medicine, Department of Occupational and Environmental Medicine Center, Linköping University, Linköping, Sweden

**Keywords:** Bystander, Harassment, Psychosocial, Conflict, Health

## Abstract

**Objective:**

This study examines under which conditions being an observer of bullying can be detrimental to health and well-being. It was hypothesized that health-related problems following observations of bullying are determined by (1) whether the observer has been exposed to bullying her/himself and (2) whether the observer have tried to intervene in the bullying situation that they witnessed.

**Methods:**

The study was based on a longitudinal probability survey of the Swedish workforce, with an 18-month time lag between assessment points (*N* = 1096).

**Results:**

Witnessing bullying at work were associated with an increase in subsequent levels of mental distress among the observers, although this association became insignificant when adjusting for the observers’ own exposure to bullying. Intervening against bullying moderated the relationship between observations of bullying and mental health problems. Observers who did not try to intervene reported a significant increase in mental health problems at follow-up, whereas there were no significant changes in levels of mental health problems among those who did intervene.

**Conclusions:**

the findings suggest that observer interventions against bullying may be highly beneficial for both the targets and observers of bullying. Organizations should therefore invest in ways to increase constructive bystander behavior in negative social situations at the workplace.

**Electronic supplementary material:**

The online version of this article (10.1007/s00420-020-01575-w) contains supplementary material, which is available to authorized users.

## Introduction

A comprehensive body of empirical evidence shows that workplace bullying is a prevalent and detrimental occupational stressor in contemporary working life (Nielsen and Einarsen 2012; Nielsen et al. 2016; Verkuil et al. 2015). With regard to the effects of bullying, those exposed report reduced health and well-being in the form of mental and somatic complaints (Finne et al. 2011; Hansen et al. 2014; Reknes et al. 2016). Exposure to bullying is also a risk factor for workability as studies with objective data have shown increased sick leave rates (Eriksen et al. 2016; Niedhammer et al. 2013; Nielsen et al. 2018) and risk for disability retirement (Glambek et al. 2015; Nielsen et al. 2017a). Due to the harmful effects of bullying on its targets, scholars have also become gradually more interested in the ripple effects on bystanders to bullying, that is, how observers react when witnessing the systematic harassment of others (e.g., Emdad et al. 2012; Totterdell et al. 2012; Vartia 2001). Some research findings indicate that observing others being bullied at work has severe negative psychological consequences for the witnesses (Hansen et al. 2013; Sims and Sun 2012; Sprigg et al. 2019). It has therefore been concluded that workplace bullying is not simply an interpersonal matter, but also a problem for the larger organization. Yet, it has also been argued that the reported consequences of observed bullying are exaggerated due to the fact that many studies on witness reactions have not taken the observers own exposure to bullying, as well as their previous levels of well-being, into consideration (Nielsen and Einarsen 2013). Hence, the actual magnitude of the consequences of observed workplace bullying on health and well-being among witnesses is still unknown. This study will contribute to the current knowledge base by using prospective data to determine whether observations of bullying is associated with subsequent changes in well-being among the observers, even when taking the observers own exposure to bullying at the workplace into account. Extending previous research, we will also examine whether intervening when witnessing bullying of others may influence the well-being of the observer.

## Health outcomes following observations of bullying at the workplace

As a workplace stressor, bullying represents an extreme form of systematic and enduring social alienation which, at least theoretically, is assumed to exceed the boundaries of other forms of interpersonal aggression such as incivility, social undermining, and verbal abuse (Tepper and Henle 2011). Formally, workplace bullying is defined as a situation in which an individual persistently and over a period of time, is on the receiving end of negative actions (i.e., bullying behaviors) from superiors and/or co-workers and where the target of the bullying may have difficulties in defending oneself against these actions (Einarsen et al. 2011; Einarsen and Skogstad 1996; Olweus 1993). Following this definition, there are three main characteristics of workplace bullying. First, an employee has to be the target of illegitimate and unwanted social behaviors in the workplace, including both verbal and non-verbal as well as active and passive negative behaviors. Secondly, the exposure must be long-lasting and relatively frequently occurring. Thirdly, the target must experience this form of mistreatment or social exclusion as a form of victimization that is so unmanageable that he or she cannot easily escape from the situation nor stop the unwanted treatment. Although there is no definitive list of bullying behaviors, workplace bullying mainly involves exposure to verbal hostility, being made the laughing stock of the department, having one’s work situation obstructed, or being socially excluded from the peer group. Empirically, such behavior has been differentiated into seven categories: work-related harassment, social isolation, attacking the private sphere, verbal aggression, the spreading of rumors, physical intimidation, and attacking personal attitudes and values (Zapf et al. 1996b).

Workplace bullying can be seen as a process comprising several phases, from the mere exposure to occasional aggressive behaviors to stages of severe victimization and trauma where the targets risk exclusion from the workplace and even from working life (Einarsen 1999). In this process, all members of the work unit may have some role, be it as perpetrator, target, active intervener, or passive onlooker (Vartia 2001). To this date, most research on bullying at the workplace has focused on targets and to some extent perpetrators, whereas the observers have received less attention (Emdad et al. 2012). Yet, there are often more potential bystanders in the actual bullying situations than there are bullies and targets (Pouwelse et al. 2018), and existing empirical evidence suggests that observations of others being bullied may have substantial negative effects also for the witnesses. Both cross-sectional and longitudinal research findings show that observed bullying is associated with mental health problems (Emdad et al. 2012; Hansen et al. 2006; Sprigg et al. 2019; Vartia 2001), sleep difficulties (Hansen et al. 2013), emotional exhaustion (Totterdell et al. 2012), and reduced job satisfaction and organizational commitment (Hauge et al. 2007; Sims and Sun 2012). In a large scale study from the UK, observers of bullying reported higher levels of health problems, sickness absenteeism, and intent to leave, and lower levels of productivity, job satisfaction, and commitment compared to non-observers (Hoel and Cooper 2000). Following these findings, it seems reasonable to put forward the preliminary hypothesis that observations of workplace bullying are negatively related to subsequent levels of well-being among the observers.

However, there are theoretical reasons for questioning such a hypothesis. The consequences of observed bullying has been explained by secondary trauma theory that assumes that observers of traumatic events, especially when they know the victim, are at greatly enhanced risk of experiencing reactions with regard to health and well-being (Emdad et al. 2013). Yet, bullying among adults usually takes the form of indirect and ambiguous behavior that is difficult to perceive and comprehend for a potential observer (Einarsen et al. 2009; Eriksen et al. 2011). According to Nielsen and Einarsen (2013), individuals other than the target him/herself may therefore only be able to perceive “snapshots” of the interactions between the target and the perpetrator and may not see the full mistreatment. Consequently, targets and observers may have quite different perceptions of the actual situation and circumstances. This is exemplified by the findings in a study of 5288 UK employees which examined how targets and observers of workplace bullying rated the leadership style of their immediate superior (Hoel et al. 2010). This study found that while observers of bullying perceived their leader as having an authoritarian leadership style, the targets of bullying viewed their superior as having a leadership style with a combination of some laissez-faire leadership and quite a bit of inconsistent punishment. As an observer of bullying will experience the nature of the actual bullying differently from the target, it seems likely that the recognition of the trauma is dependent upon the understanding of the bullying. Hence, observers that have been bullied themselves should have a more precise interpretation of the event and may thereby be more traumatized when perceiving bullying of others than non-bullied observers.

Furthermore, research has established that outcomes of exposure to bullying are dependent on the cognitive interpretation of the target, for example, the feeling of being unable to defend oneself against the mistreatment (Nielsen et al. 2012). In line with the transactional model of stress and coping (Lazarus and Folkman 1984), it is not necessarily the exposure in itself that is the biggest threat to well-being, but how the targets interpret their own ability to cope with the bullying, at least in cases of low-intense bullying (Nielsen et al. 2017b). A neutral observer of bullying is not exposed to actual harassing behaviors, and does therefore not need to cope directly with the bullying him-/herself. From this we may infer that workplace bullying should have less impact on observers compared to its targets (Nielsen and Einarsen 2013). Hence, based on the above reasoning, it seems likely that observers should be less affected by the bullying when compared to those who are actually bullied. In addition, as we will propose below, methodological issues and previously unaccounted third factors may also determine the previously reported stress reactions among observers of bullying.

With regard to methodological issues, there are strong reasons to assume that observers’ own exposure to workplace bullying is a significant confounder that can explain the magnitude of the consequences of bullying on witnesses. Previous research has found an extensive overlap between observed and self-reported exposure to bullying (Hauge et al. 2007; Hoel et al. 2010), thus indicating that many observers actually are targets of bullying themselves. Consequently, witness reports may be colored by the observer’s own exposure to bullying, and any reported outcome could be a consequence of exposure to, rather than observation of, bullying. Although there are some studies which have controlled for the observers’ own exposure to bullying (Hansen et al. 2006; Salin and Notelaers 2018; Sprigg et al. 2019), others have not (Emdad et al. 2012; Sims and Sun 2012; Totterdell et al. 2012), and it is therefore reasonable to expect that the findings of the latter studies exaggerate actual relationships. As an illustration of the importance of controlling for witnesses’ own exposure, Nielsen and Einarsen (2013) found that the association between observations of bullying and subsequent symptoms of psychological distress became insignificant when controlling for the observers self-reported exposure to bullying. This suggests that a significant part of the variation in well-being among observers of bullying could be explained by their own exposure to bullying. On the other hand, a prospective study from the UK, that controlled for the observers’ own exposure to bullying, found that witnessing the bullying of others undermined employees’ well-being (work-related depression and anxiety) 6 months later, but only if the employee were low in optimism and lacked supervisor support (Sprigg et al. 2019). Furthermore, in a cross-sectional study on the associations between witnessing workplace bullying and employee attitudes and well-being, witnessing bullying was related to work-related attitudes such as intent to leave, but not stress outcomes such as worrying and need for recovery, again when controlling for witnesses’ own experiences of bullying (Salin and Notelaers 2018). Hence, existing findings are inconsistent and more research are needed in order to understand the boundary conditions that may determine a witness’s reactions to being a bystander to bullying. Replicating previous research, the first aim of this study was therefore to investigate whether the association between observations of workplace bullying and mental health problems is influenced by the observer’s own exposure to workplace bullying. The following hypothesis will be tested:

H1: The association between observations of workplace bullying and subsequent mental health problems will attenuate when controlling for the observer’s own exposure to workplace bullying.

In order to add to the understanding of boundary conditions, and thereby extend previous research on bystanders to bullying, we will in this study also examine intervening against bullying as a conditional variable that determines when and for whom observations of bullying is detrimental. A bystander to bullying may react and act in different ways in relation to what one observes, and take on a more or less active and supportive role towards the target (Ng et al. 2019). Hence, observer outcomes of witnessing bullying may be a function of behavior and the role taken of the witness. Active defenders are those who stand up for the victims and intervene to defend and help them, whereas passive bystanders are those who are avoidant onlookers or remain a silent audience and thereby do nothing to help the victim (Salmivalli et al. 1996). In a qualitative study from India, passive bystanders to bullying experienced regrets over their limited effectiveness and struggled with confusion, guilt and remorse. They also remained emotionally disturbed with feelings of sadness, anger, guilt and fear, all typical symptoms of psychological distress (D'Cruz and Noronha 2011). Hence, whether or not a witness to bullying tries to intervene with regard to the observed bullying may influence the witnesses’ subsequent health and well-being, for example, as a result of “moral injury”. The concept of moral injury refers to an injury to an individual's moral conscience and values resulting from an act of perceived moral transgression, which produces profound emotional guilt and shame (Barnes et al. 2019; Litz et al. 2009). By doing nothing, even though they, at some level in their consciousness, acknowledge that they should or could have intervened in the situation, passive bystanders may experience emotional turmoil that shatter their moral conscience. That is, while the passive bystanders may save themselves from confronting the bully, he/she will not escape the knowledge that their lack of actions implies that the target had to continue their suffering (D'Cruz and Noronha 2011). Hence, mental health complaints may develop because of the self-guilt that follows from not intervening in the ongoing bullying. Substantiating the role of self-guilt in the development of mental complaints, meta-analytic evidence have shown that feelings of shame and guilt both are significantly associated with increased levels of psychological distress (Kim et al. 2011). Taken together, the above reasoning suggests that intervening may be an important moderator in the association between observations of bullying and health outcomes among observers in that any subsequent reported health complaint may be dependent upon whether or not the observer has tried to intervene in the bullying they witnessed:

H2: Intervening when observing others being exposed to bullying will moderate the association between observation and subsequent mental health problems in that the relationship is stronger for passive bystanders and weaker for those who intervene.

In addition to being confounded by the observer’s own exposure to bullying, and their efforts to intervene in the situation, associations between observations of workplace bullying and well-being could also be influenced by reversed causality. As most studies on observations of workplace bullying are based on cross-sectional data (Hauge et al. 2007; Sims and Sun 2012; Vartia 2001), the results do not provide information about causal relationships between the study variables. That is, although it is theoretically expected that being a witness to bullying of others leads to impaired health, it may also be that existing mental health problems influence observations of bullying. According to the “gloomy perception mechanism” (de Lange et al. 2005), distressed or dissatisfied employees may report less favorable work characteristics because they evaluate their work environment more negatively than do other employees. Consequently, compared to their more satisfied colleagues, these employees may have a lower threshold for interpreting events at the workplace as bullying due the negative perceptions that follow from distress and dissatisfaction (Nielsen and Einarsen 2013). To determine whether there is a reverse causal association between observations of bullying and mental health problems, the following hypothesis will be tested:

H3: Mental health problems are positively related to increased risk of later observations of bullying at the workplace.

## Methods

### Design and sample

The project was approved by the Regional Ethical Review Board at Linköping University, Sweden. Protocol number: 2017/336-32. The study was conducted in a probability sample of the Swedish workforce between 18 and 65 years, working at workplaces with ten or more employees, drawn by the government agency Statistics Sweden (https://www.scb.se/en). The sample was randomly selected from a population of about 3.3 million Swedish employees. This is a two-wave prospective study with a total of 1096 people responding to both waves. The study had an 18-month time lag between measurement points with questionnaire at T1 distributed in the autumn of 2017 and T2 in the spring of 2019. Synchronous effects tend to increase over time, suggesting that the effects of chronic stressors build up through cumulative exposure, and meta-analytic findings show that a time-lag of 18 months should be adequate with regard to predicting outcomes in a stressor-strain relationship (Ford et al. 2014). The response rate at T1 was 25% and 64% at T2. Only respondents who participated at T1 were invited at T2. The mean age at T1 for those responding to both waves was 49.3 years (SD = 10.0), 58% were women, 90% were born in Sweden, 54% were married, and 52% had at least one child. The mean period of employment at the current workplace was 13.5 years (SD = 11.6), 14% worked in some form of managerial position, and 96% had a fixed contract. A majority had some university or college education (60%); one third (36%) had 10–12 years of education while the rest (4%) had 9 years or less.

### Attrition analyses

Altogether 64% of the participants at baseline responded to the follow-up survey. Effects of attrition on the overall cohort was tested by comparing those who responded at both waves (the ‘stayers’) to the ones only responding to the first wave (the ‘drop-outs/lost to follow-up’). We compared a number of demographic variables (gender, age, marital status, country of birth, income, education level, and number of employees at the workplace), as well as, the main study variables (conflicting and ambiguous roles in the organization, own exposure to bullying, observation of bullying, and mental health). The analyses showed no significant differences for the majority of these variables; however, the stayers were significantly older (49 vs. 47 years), and had less mental health problems (0.6 vs. 0.7 on a scale from 0 to 3) compared to drop-outs.

### Measures

Workplace bullying was measured using the Swedish translation (Rosander and Blomberg 2018) of the Negative Acts Questionnaire–Revised (NAQ-R; Einarsen et al. 2009). NAQ-R describes 22 negative and unwanted behaviors that may be perceived as bullying if occurring on a regular basis. All items are formulated in behavioral terms and hence focus on mere exposure to inappropriate behaviors while at work with no reference to the term bullying (Einarsen and Nielsen 2015). The NAQ-R contains items referring to both direct (e.g., openly attacking the victim) and indirect (e.g., social isolation, slander) behaviors. The items also distinguish between personal and work related forms of bullying (Einarsen et al. 2009). Example items are “Being ignored or excluded”, “Repeated reminders of your errors or mistakes”, and “Someone withholding information which affects your performance”. The respondents were asked to indicate how often they had been exposed to each specific behavior at their present worksite during the last 6 months. Response categories ranged from 1 to 5 (Never, Now and then, Monthly, Weekly, to Daily). Cronbach’s alpha for NAQ-R at T1 was 0.89. In order to distinguish between targets and non-targets of bullying in analyses of prevalence, we used the previously established cut-off score of 33 on the NAQ-R (Notelaers and Einarsen 2013). In all other analyses, the NAQ-R was used as a continuous variable.

In line with the majority of previous research on the topic (see Pouwelse et al. 2018), observation of others being exposed to bullying behaviors was measured with a single item question following the NAQ–R: “Have you observed or witnessed someone being exposed to at least some of the above-mentioned negative acts during the past 6 months at your workplace?”. The answers were given on the same frequency scale as the NAQ–R. If reporting being a witness to bullying behaviors at least now and then a question about intervening followed: “If you have witnessed someone being exposed to negative acts at your workplace, have you tried to intervene?” (Yes/No). The questions about witnessing bullying are part of the PSYWEQ questionnaire (Rosander and Blomberg 2018).

Mental health was measured using the Hospital Anxiety and Depression Scale (HADS; Zigmond and Snaith 1983). HADS has 14 items using a response scale with four alternatives (0–3), for example, “I feel cheerful” with possible responses from “Not at all” to “Most of the time”. Responses were coded such as higher scores on the HADS indicate more mental health problems. A score > 14 on either of the anxiety or depression subscales indicates clinical distress (Stern 2014). Cronbach’s alpha for HADS at T1 was 0.90, and at T2 0.89.

Role conflict and role ambiguity has been established as important correlates of both workplace bullying and mental health (Finne et al. 2014; Van den Brande et al. 2016). A measure of conflicting and ambiguous roles in the organization, Roles in the Organization (RIM), taken from the PSYWEQ questionnaire (Rosander and Blomberg 2018), was therefore included as a covariate in this study. It is based on six items focusing on: (a) unclear roles, responsibilities and tasks; (b) a clear division of tasks; (c) clear roles; (d) an orderly organization; (e) well-functioning routines and organization; and (f) clear role expectations. The response scale for RIM is a seven-point Likert scale. The internal consistency in the current sample at T1 was 0.90. High values mean clear roles.

### Statistical analyses

All analyses were conducted with IBM SPSS version 26. A linear hierarchical (stepwise) regression analysis (ordinary least square) was used to test H1. For H2, we conducted a moderation analysis using Hayes PROCESS macro (version 3.4) for SPSS (Hayes 2012, 2018). PROCESS uses an ordinary least squares or logistic regression-based path analytical framework for estimating direct and indirect associations in two and three way interactions in moderation models along with simple slopes and regions of significance for probing interactions (see https://www.afhayes.com for further description and documentation). All continuous scale variables were mean-centered in the analyses of interaction effects. Finally, a logistic regression analysis was used to test H3. For all three hypotheses age, gender, and roles in organization at T1 were covariates. For H2 also HADS at T1 was added as covariate. H3 were also adjusted for witnessing of bullying at baseline.

## Results

**Fig. 1:**
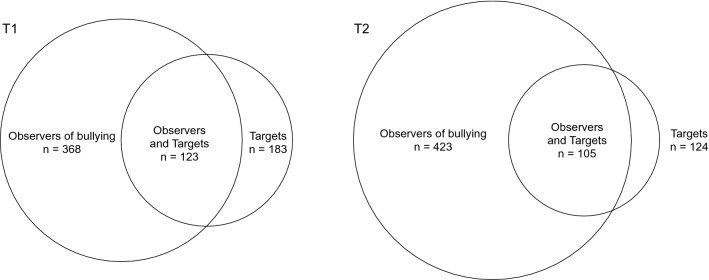
Venn diagram of participants observing others being bullied, participants that were targets, and the overlap of those both observing others and being a target themselves

At baseline, 33% of the sample had observed others being bullied at their current workplace at least “now and then” or more often, while 17% were targets of bullying when following the wider cut-off value of 33 on the NAQ-R. At follow-up, 40% had observed bullying “now and then” or more often, while 12% were estimated to be targets of bullying. Altogether 21% of the total sample were observers of bullying at both baseline and follow-up, whereas 7% of the total sample were targets of bullying at both measurement points. At baseline 33% of those who had observed bullying of others “now and then or more often” reported to be a target of bullying themselves. The corresponding number at follow-up was 25%. Altogether 50% of those who had observed bullying of others had intervened against the bullying. Figure 1 presents Venn diagrams for participants observing others being bullied, participants that were targets, and the overlap of those both observing others and being a target themselves at baseline and follow-up.

Means, standard deviations and inter-correlations for all study variables are presented in Table 1. In the following analyses of observation of bullying, all positive responses (i.e., now and then or more often) were recorded into an “observer” category in order to increase the statistical power. The respondents’ own exposure to bullying was significantly correlated with observing bullying both at baseline (*r* = 0.51; *p* < 0.001) and follow-up (*r* = 0.32; *p* < 0.001). A logistic regression analysis further showed that the respondents’ own exposure to bullying behavior at baseline was significantly associated with observations of bullying at follow-up (OR = 4.82; 95% CI 2.63 to 8.82) when controlling for age, gender, and roles in organization (Table 2). This association between own exposure to bullying and subsequent observations of others being bullied remained significant also after adjusting for observations of bullying at T1.

**Table 1:** Means, standard deviations (SD) and Pearson inter-correlations for all measures used in the study

	*n*	Mean	SD	1.	2.	3.	4.	5.	6.	7.	8.
1. Sex	1096	0.58	0.49								
2. Age	1096	49.30	10.05	0.00							
3. Roles in the organization (T1)	1096	5.14	1.29	0.07*	0.11**						
4. Exposure to negative acts (T1)	1094	1.25	0.33	– 0.06*	– 0.11**	– 0.43**					
5. Mental health (T1)	1092	0.64	0.47	0.08*	– 0.12**	– 0.040**	0.48**				
6. Mental health (T2)	1084	0.61	0.45	0.07*	– 0.12**	– 0.26**	0.35**	0.68**			
7. Witnessing (T1)	1087	1.44	0.75	0.01	– 0.08**	– 0.33**	0.51**	0.32**	0.24**		
8. Witnessing (T2)	1062	1.50	0.73	0.04	– 0.18**	– 0.22**	0.32**	0.24**	0.29**	0.37**	
9. Intervening (T1)	349	0.52	0.50	0.03	0.07	– 0.06	0.04	– 0.04	– 0.09	– 0.09	– 0.03

Reference category for sex is “female”. All other variables are continuous

*p < 0.05, **p < 0.001

**Table 2:** Logistic regression analysis prediction witnessing bullying at follow-up (χ^2^ = 203.42, *p* < 0.001)

	OR	95% CI	*p* value
Exposure to negative acts (T1)	4.82	[2.63; 8.82]	< 0.001
Age	0.98	[0.96; 0.99]	< 0.001
Sex	1.34	[1.01; 1.77]	< 0.05
Roles in the organization (T1)	0.88	[0.78; 0.98]	< 0.05
Witnessing (T1)	2.24	[1.75; 2.86]	< 0.001

Reference category for sex is “female”. All other variables are continuous

*OR* odds ratio, *CI* confidence interval

Associations between observed bullying at baseline and mental health problems at follow-up are presented in Table 3. In step 1, baseline observations of bullying significantly predicted an increase in mental health problems at follow-up (*ß* = 0.16; *p* < 0.001) after adjusting for age (*ß* = − 0.08; *p* < 0.01), gender (*ß* = 0.08; *p* < 0.01), and roles in the organization (*ß* = − 0.19; *p* < 0.001). In line with our first hypothesis, this association between observed bullying and mental health problems became insignificant (*ß* = 0.06; *p* > 0.05) after controlling for the respondents’ own exposure to bullying (*ß* = 0.25; *p* < 0.001) in the second step of the regression. Hence, the association between observations of workplace bullying and mental health problems is attenuated when controlling for the observers own exposure to workplace bullying. Baseline mental health complaints (*ß* = 0.66; *p* < 0.001) were included in the third and final step of the regression. None of the bullying variables were significantly associated with mental health problems at follow-up when adjusting for baseline mental health, thus indicating that previous mental health problems is the most prominent predictor of later mental health problems. A sensitivity analyses that excluded respondents with HADS-scores at baseline above the clinical cut-off value of 14 on either the anxiety or depression subscales, or on both (*N* = 30), replicated the findings from the main analysis, thus showing that observations of workplace bullying was not associated with changes in mental health problems when adjusting for the respondents’ own exposure to bullying.

**Table 3:** Hierarchical regression analysis predicting mental health at T2 (H1)

	*b*	SE *b*	95% CI *b*	*ß*	*R*^2^	∆*R*^2^	*F*
Step 1					0.10		30.54***
Witnessing (T1)	0.10	0.02	[0.06; 0.13]	0.16***			
Age	− 0.00	0.00	[− 0.00; − 0.00]	− 0.08**			
Sex	0.08	0.03	[0.03; 0.13]	0.08**			
Roles in the organization (T1)	− 0.07	0.01	[− 0.09; − 0.05]	− 0.19***			
Step 2					0.14	0.04***	35.81***
Witnessing (T1)	0.04	0.02	[− 0.00; 0.08]	0.06			
Age	− 0.00	0.00	[− 0.00; 0.00]	− 0.07*			
Sex	0.09	0.03	[0.04; 0.14]	0.10***			
Roles in the organization (T1)	− 0.04	0.01	[− 0.06; − 0.02]	− 0.12***			
Exposure to negative acts (T1)	0.34	0.05	[0.25; 0.44]	0.25***			
Step 3					0.46	0.32***	150.88***
Witnessing (T1)	0.01	0.02	[− 0.02; 0.04]	0.01			
Age	− 0.00	0.00	[–0.00; 0.00]	− 0.03			
Sex	0.02	0.02	[− 0.02; 0.06]	0.02			
Roles in the organization (T1)	0.01	0.01	[− 0.01; 0.03]	0.02			
Exposure to negative acts (T1)	0.04	0.04	[− 0.04; 0.12]	0.03			
Mental health (T1)	0.64	0.02	[0.58; 0.68]	0.66***			

Dependent variable: Mental health (T2). Reference category for sex is “female”. All other variables are continuous

*b* unstandardized coefficient, *ß* standardized coefficient, *CI* confidence interval

*p < 0.05, **p < 0.01, ***p < 0.001

**Fig. 2:**
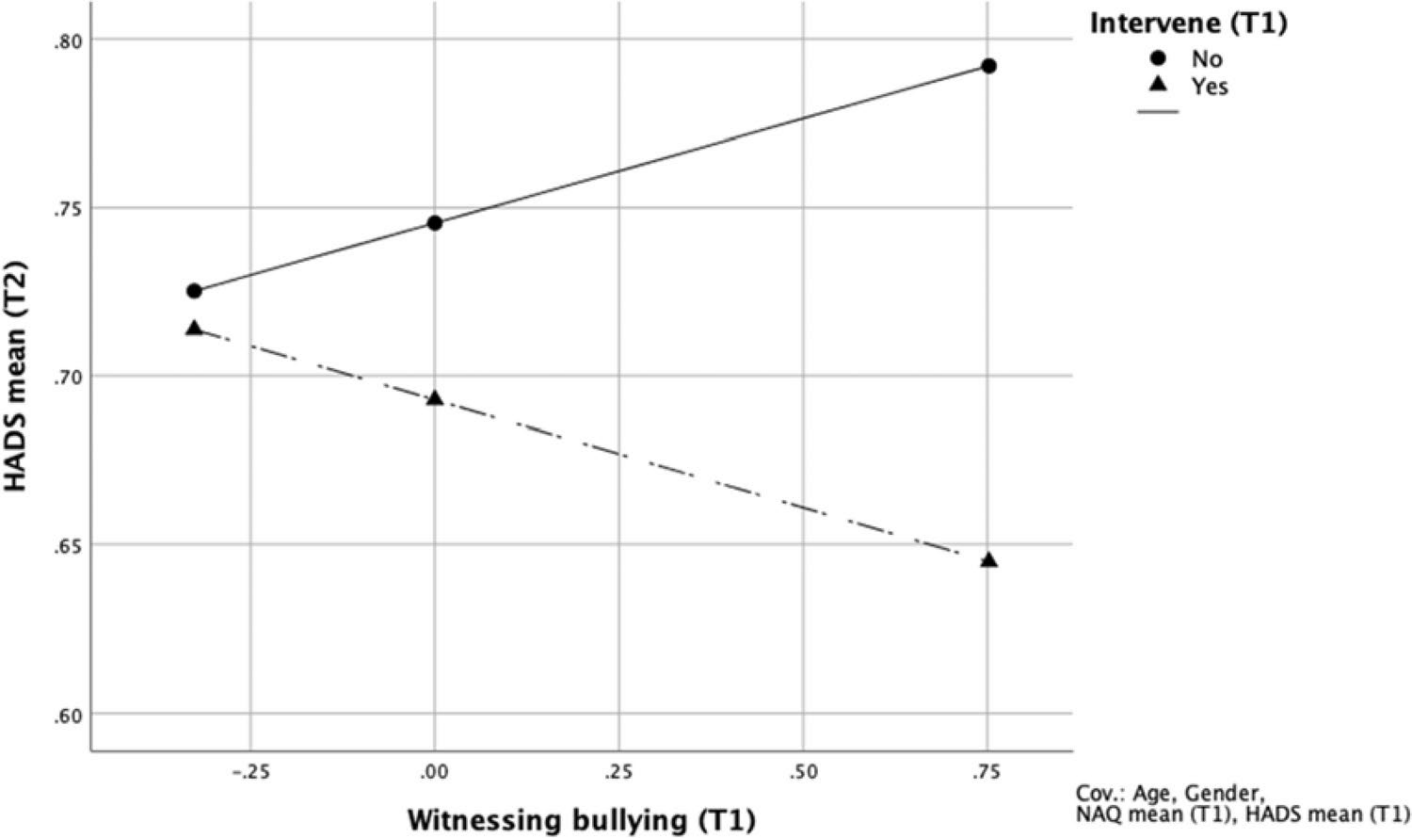
The interaction between witnessing bullying and intervening at T1 with regard to mental health at T2 (H2). Scores for the independent variable plotted at − 1 SD, mean, and + 1 SD (Mean-centered)

Testing our second hypothesis, a follow-up regression analyses was conducted to determine the interactive effects between observed bullying and the observer’s effort to intervene with regard to the observer’s mental health (Table 4). Only respondents that had observed bullying of others were included in this analysis (*N* = 343) and the indicator of observed bullying was treated as an ordinal variable in this analysis. After adjusting for age, gender, exposure to bullying, roles in organization, and baseline mental health problems, the findings showed that efforts to intervene moderated the association between observations of bullying at baseline and levels of mental health problems at follow-up (*b* = − 0.13; 95% CI − 0.23 to − 0.02). A simple slope tested revealed that observers who did not try to intervene report a significant increase in mental health problems at follow-up (*b* = 0.06; 95% CI − 0.00 to − 0.13), whereas the changes in levels of mental health problems among those who did intervene was insignificant (*b* = − 0.06; 95% CI − 0.15 to − 0.03). The results are graphically displayed in Fig. 2. A sensitivity analysis that excluded respondents with clinical scores on the HADS at baseline, replicated the above findings.

**Table 4:** Moderation analysis predicting mental health at T2 (H2)

	*b*	SE *b*	95% CI *b*	*p* value
Witnessing (T1)	0.06	0.03	[− 0.00; 0.13]	0.064
Intervene (T1)	− 0.05	0.04	[− 0.13; 0.02]	0.171
Witnessing (T1) × intervene (T1)	− 0.13	0.05	[− 0.23; − 0.02]	0.020
Sex	0.01	0.04	[− 0.08; 0.07]	0.823
Age	0.00	0.00	[− 0.00; 0.00]	0.346
Exposure to negative acts (T1)	− 0.01	0.06	[− 0.13; 0.12]	0.923
Roles in the organization (T1)	0.00	0.02	[− 0.03; 0.03]	0.880
Mental health (T1)	0.64	0.05	[0.55; 0.73]	< 0.001

*N* = 343 (*F* = 35.01; *p* < .001). Dependent variable: Mental health (T2). Reference category for sex is “female”. All other variables are continuous

*b* unstandardized coefficient, *CI* confidence interval

Our third hypothesis proposed that mental health problems would be positively related to an increased risk of later observations of bullying at the workplace*.* This analysis included all respondents that had provided answers to the indicator of observations of bullying at T2 (*N* = 1050). There was a positive correlation between mental health problems at baseline and witnessing bullying at follow-up (*r* = 0.24; *p* < 0.001). A logistic regression analysis showed a significant association between baseline mental health problems and subsequent observations of bullying at follow-up (OR = 1.85; 95% CI 1.34 to 2.55) controlling for age, gender, and roles in organization, as well as adjusting for observation of bullying at baseline (Table 5). A sensitivity analysis that excluded respondents that had observed bullying of others at baseline “now and then” or more often (*N* = 346), replicated the above findings. Detailed findings from the sensitivity analyses can be obtained by contacting the first author.

**Table 5:** Logistic regression analysis prediction witnessing bullying at follow-up (χ^2^ = 191.07; *p* < .001)

	OR	95% CI	*p* value
Mental health problems (T1)	1.85	1.34; 2.54	< 0.001
Age	0.98	0.96; 0.99	< 0.001
Sex	1.21	0.92; 1.60	> 0.05
Roles in the organization (T1)	0.84	0.74; 0.94	< 0.01
Witnessing (T1)	2.58	2.04; 3.27	< 0.001

Reference category for sex is “female”. All other variables are continuous

*OR* odds ratio, *CI* confidence interval

## Discussion

Some previous research findings indicate that the mere observation of others being bullied at the workplace have negative health consequences for the observer (Emdad et al. 2012). Questioning this finding, other scholars have shown that this association between observations of bullying and mental health problems disappears when adjusting for the observers own exposure to workplace bullying, thus indicating that the reported health problems among observers actually are due to personal experiences with bullying (Nielsen and Einarsen 2013; Sprigg et al. 2019). The present study replicated this latter finding by showing that the association between observations of bullying and mental health complaints became insignificant when adjusting for the observers own exposure to bullying. However, extending previous research, a novel and important finding of this study was that the association between observations of bullying and health problems is dependent upon whether or not the observer tried to intervene in the bullying they witnessed. That is, observers who did not try to intervene reported a significant increase in mental health problems at follow-up, even when controlling for their own exposure to bullying, whereas there was no association between observations of bullying and mental health problems among observers who tried to intervene and stop the bullying. Furthermore, we also established a significant association between existing mental health complaints and observations of bullying as respondents with such complaints had an increased likelihood of observing new cases of bullying. The results were replicated in a series of sensitivity analyses, thus indicating the validity of the findings. Taken together, the findings supported all three study hypotheses.

In line with findings from some previous studies on bystanders to bullying (Nielsen and Einarsen 2013; Sprigg et al. 2019), our results suggest that a failure to partial out the effects of the observer’s own exposure to bullying will confound the effects of witnessing bullying due to a large overlap between observed and experienced bullying. This means that previous findings that have showed an increase in health problems among the observers after witnessing bullying may simply be caused by the fact that many witnesses themselves have been exposed directly to bullying. However, the finding that intervening in the bullying of others act as a moderator variable nuances this understanding of how bystanders to bullying are impacted by their observations. In line with previous findings on conditional factors (Sprigg et al. 2019), our findings indicate that observing bullying can be detrimental for the observer irrespective of his/her previous exposure, but only under specific conditions. That is, witnessing bullying of others seems to be related to increased mental health problems when observes do not try to intervene in the perceived bullying. Observers that try to intervene experience no subsequent health problems. This impact of intervening on the association between observation of bullying and subsequent increase in mental health problems was significant even after adjusting for existing levels of mental health and the experience of conflicting and ambiguous roles at the workplace. Hence, it seems unlikely that the association between observations of bullying and mental health is due to poorer conditions in the respondents’ organizations.

As discussed in the introduction, witnessing bullying in itself do not constitute a health risk, but rather the observer’s moral judgement of their own conduct in the situation. Employees know that bullying is inappropriate and that the target is likely to suffer. Owing to their internal moral obligations, most humans consider themselves as benevolent and responsible persons (Janoff-Bulman 1992), the knowledge that one did not try to help a bullied colleague may thereby lead to feelings of shame and guilt due to an injury to the individual's moral conscience and values. Experiencing dissonance between self-perceptions and actual behavior over a prolonged time may subsequently develop into psychological distress in the form of anxiety and depression (Mikkelsen 2001). This means that helping behavior is crucial with regard to understanding how workplace bullying can influence observers, and future research should therefore address when and under which conditions bystanders intervene as well as the dynamics of bystander behavior over time (Mulder et al. 2017; Ng et al. 2019).

While it has been argued that mental health complaints should increase the likelihood of observing bullying of others at the workplace, due to more negative perceptions of the work environment (Nielsen and Einarsen 2013), previous research has provided mixed findings with regard to whether there is a such a reverse relationship between observations of bullying and mental health problems. Whereas Nielsen and Einarsen (2013) found baseline symptoms of psychological distress to predict observing new cases of bullying at follow-up in sample of Norwegian employees, Sprigg and colleagues (2019) found no effects of depression, anxiety, or emotional exhaustion on risk for later observations of bullying in their longitudinal study of UK workers. In the current study, we found mental health complaints at baseline to be associated with a significantly higher risk (OR = 1.86; 95% CI 1.34 to 2.56) of subsequent observations of new cases of bullying (i.e., after adjusting for previous observations). Hence, our findings indicate that employees with existing mental health complaints are more likely to observe bullying of others compared to employees without such complaints. However, more research is needed to further establish the nature of this association.

### Methodological strengths and limitations

This study has some notable strengths. Extending previous cross-sectional studies from small convenience studies (Hellemans et al. 2017; Mulder et al. 2017; Sims and Sun 2012), we examined prospective associations between observations of bullying, own exposure to bullying, measures to intervene, and mental health problems using time-lagged data. The sample was drawn from the total pool of Swedish employees and is a national probability sample of Swedish workers. However, whereas the response rate at T2 was adequate (64%), the baseline response rate of 25% was somewhat lower than average for this kind of surveys (Baruch and Holtom 2008). However, as there seem to be a strong secular trend of reduction in survey response rates in recent years, the response obtained in the present study may not actually be deviating from the current average (Stedman et al. 2019). Furthermore, although the low response rate at T1 may influence the external validity of the findings of the study, response rate should little impact on the internal validity (Schalm and Kelloway 2001). With regard to the attrition from baseline to follow-up, analyses showed that stayers were significantly older, and had less mental health problems, compared to drop-outs. This may indicate a healthy worker bias on participation at follow-up in that the healthy workers seems to be more likely than unhealthy workers to participate. Although previous research have shown that the health status of participants at baseline seems to have little impact on the external and internal validity of the follow-up assessment in prospective survey research (Nielsen and Knardahl 2016), this bias should be considered in the interpretation of the current study.

Regarding possible limitations, all measurement instruments were self-report measures. Hence, biases such as response set tendencies, social desirability and common method variance may have influenced our findings (Podsakoff and Organ 1986). Yet, the use of a time lag of 18 months between the measurement of the independent and dependent variables should reduce the latter risk (Podsakoff et al. 2003). It should be noted that other findings may have been obtained with different time lags (Ford et al. 2014). For instance, and as noted above, there may be important “sleeper effects”, that is, the effects appear a long time after exposure to the stressor (Zapf et al. 1996a). As argued by Taris and Kompier (2014), reporting a non-significant finding based on the use of too short or too long time intervals may conceal a true causal tendency.

Observations of bullying and intervening when observing bullying were both assessed with single item questions. Whereas the use of single item questions is the common measurement approach in research on observations of bullying (Pouwelse et al. 2018), single item questions may not fully capture all aspects of the assessed phenomena. Hence, although while there is a wide variety of different possible bystander behaviors (Ng et al. 2019), bystander response was reduced to an active and passive dichotomy in the current study. The results of the current study could therefore have been more informative if a more detailed checklist was applied. Nonetheless, there are also multiple advantages with the use of single item questions, such as cost-efficiency, greater face validity, and the increased willingness of respondents to take the time to complete the questionnaire when the number of items is reduced. Single-item measures can be reliable, as estimated by test–retest correlations (Littman et al. 2006), correlate strongly with multiple-item scales (Wanous et al. 1997), and can predict outcomes effectively (Nagy 2002).

## Conclusions and implications

We have examined whether and under which conditions workplace bullying can influence health and well-being of bystanders. Extending previous research on this issue, our prospective study shows that witnessing bullying of others at one’s workplace can be detrimental for the observer’s mental health, yet only in cases where the observer remains passive and do not try to intervene in the bullying. Hence, our findings add to a growing body of research on the importance of understanding the impact of merely witnessing the predicament of others at work (Sprigg et al. 2019). This finding has important theoretical, methodological, and practical implications. With regard to theory, our results support theoretical models of bystander that highlights the actual behavior and reactions of non-bullied third parties as important for understanding the bystander phenomenon (Ng et al. 2019). However, as we did not examine the impact of different bystander roles, or how the observers actually intervened against the bullying, future research on observers of bullying should have a more in debt focus on different bystander roles and specific forms of helping behavior they display. With regard to methodology, the study findings imply that upcoming research on witnesses to bullying need to include measures of intervening as a study variable in order to fully understand the effects of being a bystander.

However, the practical implications of our findings may be the most important. First, intervening against bullying may be highly beneficial for the target of the bullying. If intervening contributes to end the bullying, the target may be saved from detrimental long-term health consequences (Nielsen et al. 2015a, b). Yet, even if the attempt at intervening fails, the target will experience social support, something that in itself may buffer the negative effects of bullying (Nielsen et al. 2019). Second, as shown by our findings, intervening when witnessing bullying of others is a moral decision that will also reduce the detrimental effects of bullying on the observer. Hence, by intervening against the observed bullying, a bystander can “kill two birds with one stone”. This means that employers and organizations may benefit from investing in ways to increase active and constructive bystander behavior in negative social situations at work and to prevent risk situations for bullying (Pouwelse et al. 2018). Developing and evaluating such bystander interventions is therefore a highly important area for practitioners and researchers in the years to come.

## Electronic supplementary material

Below is the link to the electronic supplementary material.

Additional file1 (DOCX 20 kb)
