# Supplementary material for: Killing two birds with one stone: how intervening when witnessing bullying at the workplace may help both target and the acting observer
Source: Int Arch Occup Environ Health. 2020 Sep 14;94(2):261–73. doi: 10.1007/s00420-020-01575-w (PMC7872954; doi:10.1007/s00420-020-01575-w)
Supplement: Supplementary file 1 — Additional file1 (DOCX 20 kb) [file 420_2020_1575_MOESM1_ESM.docx]

**Results from the sensitivity analyses**

Table 3b.  *Hierarchical regression analysis predicting mental health at T2 (H1)*

|  | b | SE b | 95% CI b | ß | R^2^ | ∆R^2^ | F |
| --- | --- | --- | --- | --- | --- | --- | --- |
| *Step 1* |  |  |  |  | .09 |  | 25.02*** |
| Witnessing (T1) | 0.08 | 0.02 | [0.05; 0.12] | 0.14*** |  |  |  |
| Age | –0.00 | 0.00 | [–0.00; –0.00] | –0.09** |  |  |  |
| Sex | 0.07 | 0.02 | [0.02; 0.12] | 0.08** |  |  |  |
| Roles in the organization (T1) | –0.06 | 0.01 | [–0.08; –0.04] | –0.18*** |  |  |  |
| *Step 2* |  |  |  |  | .12 | .03*** | 33.05*** |
| Witnessing (T1) | 0.04 | 0.02 | [–0.00; 0.07] | 0.06 |  |  |  |
| Age | −0.00 | 0.00 | [−0.00; –0.00] | −0.08** |  |  |  |
| Sex | 0.08 | 0.02 | [0.03; 0.13] | 0.09** |  |  |  |
| Roles in the organization (T1) | −0.04 | 0.01 | [−0.06; −0.02] | −0.13*** |  |  |  |
| Exposure to negative acts (T1) | 0.28 | 0.05 | [0.18; 0.37] | 0.20*** |  |  |  |
| *Step 3* |  |  |  |  | .41 | .29*** | 119.13*** |
| Witnessing (T1) | 0.01 | 0.02 | [−0.02; 0.04] | 0.01 |  |  |  |
| Age | –0.00 | 0.00 | [–0.00; 0.00] | −0.03 |  |  |  |
| Sex | 0.02 | 0.02 | [−0.02; 0.06] | 0.03 |  |  |  |
| Roles in the organization (T1) | 0.00 | 0.01 | [−0.01; 0.02] | 0.02 |  |  |  |
| Exposure to negative acts (T1) | 0.03 | 0.04 | [−0.05; 0.11] | 0.02 |  |  |  |
| Mental health (T1) | 0.63 | 0.03 | [0.58; 0.69] | 0.62*** |  |  |  |

Notes: Dependent variable: Mental health (T2); b: Unstandardized coefficient; ß: Standardized coefficient; CI: Confidence Interval. Reference category for sex is “female”. All other variables are continuous.

* p < .05, ** p < .01, *** p < .001

Table 4b. *Moderation analysis predicting mental health at T2 (H2).* N=329 (F = 24.48, p < .001)

|  | b | SE b | 95% CI b |  |
| --- | --- | --- | --- | --- |
| Witnessing (T1) | 0.07 | 0.04 | [–0.00; 0.14] | p = .058 |
| Intervene (T1) | –0.07 | 0.04 | [–0.14; 0.01] | p = .094 |
| Witnessing (T1) x Intervene (T1) | –0.14 | 0.06 | [–0.26; –0.03] | p = .017 |
| Sex | –0.01 | 0.04 | [–0.09; 0.07] | p = .776 |
| Age | –0.00 | 0.00 | [–0.00; 0.00] | p = .454 |
| Exposure to negative acts (T1) | –0.02 | 0.06 | [–0.15; 0.11] | p = .739 |
| Roles in the organization (T1) | 0.00 | 0.02 | [–0.03; 0.03] | p = .957 |
| Mental health (T1) | 0.61 | 0.05 | [0.51; 0.71] | p < .001 |

Notes: Dependent variable: Mental health (T2); b: Unstandardized coefficient; CI: Confidence Interval. Reference category for sex is “female”. All other variables are continuous.

Table 5b. *Logistic regression analysis prediction witnessing bullying at follow-up (H3) (χ*^2^ = 48.58, p < .001)

|  | OR | 95% CI |  |
| --- | --- | --- | --- |
| Mental health problems (T1) | 1.90 | [1.26; 2.86] | p = .002 |
| Age | 0.98 | [0.96; 0.99] | p = .005 |
| Sex | 1.30 | [0.91; 1.86] | p = .148 |
| Roles in the organization (T1) | 0.76 | [0.65; 0.88] | p < .001 |

Notes: OR: Odds Ratio; CI: Confidence Interval. Reference category for sex is “female”. All other variables are continuous.
